# Schistosoma Japonicum UDP-Glucose 4-Epimerase Protein Is Located on the Tegument and Induces Moderate Protection against Challenge Infection

**DOI:** 10.1371/journal.pone.0042050

**Published:** 2012-07-27

**Authors:** Pingping Liu, Yanli Shi, Yunxia Yang, Yufan Cao, Yaojun Shi, Hao Li, Jinming Liu, Jiaojiao Lin, Yamei Jin

**Affiliations:** 1 National Laboratory of Animal Schistosomiasis Control/Key Laboratory of Animal Parasitology, Ministry of Agriculture, Shanghai Veterinary Research Institute, Chinese Academy of Agricultural Sciences, Shanghai, People’s Republic of China; 2 College of Animal Science and Technology, Northeast Agricultural University, Harbin, Heilongjiang Province, People’s Republic of China; Instituto Butantan, Brazil

## Abstract

Schistosomiasis is an important global public health problem, as millions of people are at risk of acquiring this infection. An ideal method for sustainable control of schistosomiasis is using a vaccine alone or in combination with drugs. In the present study, we cloned the SjGALE gene and generated the expression product in *E. coli*. The expression level of SjGALE during different developmental stages of *S. japonicum* was evaluated by real-time RT-PCR and western blotting. Immunolocalization indicated that the protein was mainly located on the tegument of the parasite. Infection of rSjGALE-immunized mice demonstrated a 34% and 49% reduction of the mean worm burden and liver egg burden, respectively, in two independent experiments, indicating immune protection. The liver egg count from each female adult worm was significantly reduced by 63% in the two trials. The cytokine profile and IgG isotype analysis demonstrated the induction of a Th1 immune profile in response to immunization with this protein, further suggesting protection against infection. In conclusion, these findings indicated that SjGALE is a potential vaccine against *S. japonicum*.

## Introduction

Schistosomiasis is an important helminth infection and mainly occurs in developing countries. Despite decades of control, there are still millions of people at risk of contracting this infection [1]. Current schistosomiasis control strategies are mainly based on safe and effective drugs, such as praziquantel and oxamniquine, but these do not prevent reinfection and the number of infected people has remained constant [2], [3]. The best long-term strategy to control schistosomiasis is through immunization with an anti-schistosomiasis vaccine combined with drug treatment [4]. A vaccine that induces even a partial reduction in worm burdens could considerably reduce pathology and limit parasitic transmission [5]. Recently, novel potential vaccine antigens were evaluated, but the level of protection obtained by vaccination with these antigens rarely exceeded the 40% benchmark set by the The World Health Organization (WHO) [6]. Therefore, it is necessary to search for alternative highly protective vaccine candidates.

In all organisms, galactose metabolism is catalyzed by three enzymes: galactokinase, galactose-1-phosphate uridyltransferase, and uridine diphosphate (UDP) galactose 4′-epimerase (GALE) [7]–[9]. GALE catalyzes the interconversion of UDP-galactose and UDP-glucose during normal galactose metabolism and it tightly binds the co-factor nicotinamide adenine dinucleotide (NAD^+^) required for catalytic activity [10]. GALE also plays a pivotal role in the formation of extracellular polymeric substance (EPS), lipopolysaccharide (LPS), and capsular polysaccharide (CPS), which are related to biofilm formation [11]–[13]. In humans, GALE deficiency results in an inborn error of metabolism, galactosemia [7]. GALE also play an important role in the development of *Drosophila melanogaster* and *Trypanosoma brucei* [14], [15]. The gene encoding GALE also exists in *Schistosoma japonicum* (SjGALE) [16], [17]; however, its specific function has not been elucidated.

In the present study, we cloned and expressed full-length SjGALE cDNA and analyzed its expression level at different stages of schistosomal developmental and the localization of the protein. We also evaluated this protein as a vaccine candidate in vivo by examining the SjGALE-induced humoral and cellular immune protective mechanisms in a mouse model of schistosomal infection.

## Materials and Methods

### Ethics Statement

All animal care and procedures were conducted according to the guidelines for animal use in toxicology (Society of Toxicology USP, 1989). The study protocol was approved by the Animal Care and Use Committee of the Shanghai Veterinary Research Institute, Chinese Academy of Agricultural Sciences. The approval ID number is:SYXK 2011-0116.

### 1. SjGALE Cloning and Molecular Characterization

The 5′ and 3′ oligonucleotides, CG GGA TCC ATG CAG AAA GGT GAT AAA GGA and CC CTC GAG TCA ATT ATT TTC AGA ATT TAT (*Bam*HI and *Xho*I sites are underlined), were used to amplify the complete SjGALE open reading frame (ORF) (GenBank accession SJCHGC06074). The polymerase chain reaction (PCR)-generated fragment was cloned into the pMD18-T vector and sequenced to confirm its identity.

Signal peptide prediction was performed using the SignalP 3.0 server (http://www.cbs.dtu.dk/services/SignalP/) and N-glycosylation sites were analyzed using the NetNGlyc 1.0 server (www.cbs.dtu.dk/services/NetNGlyc/). Transmembrane helices were analyzed using TMHMM server version 2.0 (http://www.cbs.dtu.dk/services/TMHMM-2.0/). The molecular weight (MW) and isoelectric point (pI) were calculated using the Compute pI/Mw tool (http://www.expasy.ch/tools/pitool.html). The amino acid sequences of the GALE protein were obtained from GenBank and were aligned using ClustalX software (http://www.clustal.org/). The Conserved Domain (CD)-Search algorithm (http://www.ncbi.nlm.nih.gov/Structure/cdd/cdd.shtml) was used to find conserved epimerase domains.

### 2. Expression of Recombinant Protein and Polyclonal Antibody Production

The full-length cDNA was subcloned using the enzymes *Bam*HI and *Xho*I and the pET28 vector to produce a protein that contained an N-terminal hexahistidine tag. The recombinant pET28-SjGALE plasmid construction was overexpressed in BL21 (DE3) cells (Tiangen Biotech Co., Ltd., Beijing, China) using 1 mM of isopropyl-β-D-thiogalactopyranoside (IPTG) at 37°C for 4–5 h. The bacteria were then harvested by centrifugation at 10000 rpm for 15 min. The pellet was suspended in 50 mL of phosphate buffered saline (PBS, pH 7.4) and extracted using an ultrasonic processor to release the fusion proteins, which were purified using a Ni-NTA His-Bind Resin (Qiagen GmbH, Hilden, Germany). The purified recombinant protein was injected into 5 BALB/c mice to produce polyclonal antibodies, which were used for western blotting and immunolocalization.

### 3. Real-time RT-PCR Analysis

Total RNA was extracted from *S. japonicum* at different life cycle stages using the RNeasy Protect Mini Kit (Qiagen), per the manufacturer’s instructions. cDNA was synthesized using SMART-Scribe reverse transcriptase (Clontech Laboratories, Inc., Mountain View, CA, USA) according to standard protocols. Reaction conditions were as described in the SYBR green kit and the cycling conditions were as follows: 95°C for 15 min followed by 40 cycles of 95°C for 15 s, 58°C for 15 s, and 72°C for 20 s. The generation of a specific PCR product was also tested using melting curve analysis. The independent experiments were repeated three times, using β-tubulin as an endogenous standard for each sample. Quantitation of relative differences in expression was calculated using Realplex software (Eppendorf, Hamburg, Germany).

### 4. Western Blot Analysis

All parasites were collected using tris buffer (pH 7.8), and worms were homogenized and sonicated five times for 10 s each with an interval of 15 s and centrifuged at 12000 × g for 40 min at 4°C. The supernatant was collected and protein concentrations were determined with a BCA Protein Assay Kit (Beyotime Institute of Biotechnology, Haimen, China). Protein extracts (40 µg) of each stage were then subjected to 12% sodium dodecyl sulfate polyacrylamide gel electrophoresis (SDS-PAGE), transferred to a nitrocellulose membrane (Millipore, Billerica, MA, USA), and blocked with PBS with 0.05% Tween 20 (PBS-T) plus 3% bovine serum albumin (BSA) at 4°C overnight. The membranes were washed three times with PBS-T and probed with anti-SjGALE mice serum diluted 1∶200 or anti-tubulin primary antibody (Beyotime Institute of Biotechnology) diluted 1∶1000 in PBS-T for 1 h at room temperature (RT). Then, the membranes were washed three times and probed with anti-mouse IgG conjugated to horse radish peroxidase (HRP) diluted 1∶10000 in PBS-T for 1 h. After three washes, the membranes were developed using enhanced chemiluminescence (ECL) substrate (Thermo Scientific - Pierce Protein Biology Products, San Diego, CA, USA) and imaged using the Imagequant LAS 4000 mini biomolecular imager (GE Healthcare, Waukesha, WI, USA). The western blot bands were converted into a histogram by measuring the optic density of the autoradiogram bands using Image J software (http://rsbweb.nih.gov/ij/).

### 5. Immunolocalization

Freshly perfused adult worms of *S. japonicum* were embedded in optimal cutting temperature (OCT) compound medium and pre-cooled in freezing microtome cryostat for 30 min, then 8 µm sections were prepared for assays. Slices were fixed with pre-cooled acetone for 5 min and the sections were then immunolabeled using indirect immunofluorescence as follows: parasites were blocked with 10% goat serum in PBS for 1 h at RT and incubated with anti-rSjGALE serum diluted 1∶50 in blocking buffer overnight at 4°C. Serum from non-immunized mice was used as a negative control. Samples were washed three times with PBS-T and incubated with FITC (fluorescein isothiocyanate)-conjugated anti-mouse IgG antibody (Invitrogen) diluted 1∶1000 in blocking buffer for 30 min at RT. Sections were then washed three times in PBS-T and counterstained with 0.1 mg/mL DAPI (4′,6-diamidino-2-phenylindole), which stains nuclei. The parasites were visualized using a Nikon D-ECLIPSE C1 confocal microscope system (Nikon Instruments Inc., Melville, NY, USA).

### 6. Immunization of Mice

Six to eight week-old male BALB/c mice were divided into two groups of 10 mice each. Animals were subcutaneously injected with 50 µg of rSjGALE fusion protein on days 0, 15, and 30. The recombinant protein was formulated with complete Freund’s adjuvant (CFA) for the first immunization and incomplete Freund’s adjuvant (IFA) for the boost. In the control group, adjuvant in PBS was administered using the same immunization protocol.

**Figure 1:**
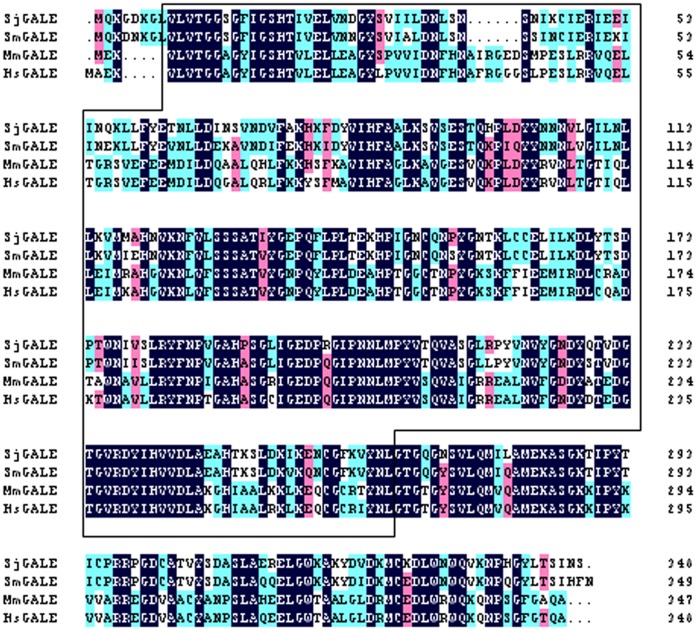
The complete protein sequence of SjGALE in relation to *S. mansoni*, mouse and human GALE. Clustal X alignment of the derived amino acid sequences of SmGALE (XP 002578272.1), MmGALE (NP_848476.1), and HsGALE (NP 001008217). The regions with high identity and similarity between GALE sequences are shown in color. The epimerase domain (continuous box), active sites (#) and NAD binding sites (*) are also indicated.

### 7. Challenge Infection and Worm Burden Recovery

Two weeks after the last boost, mice were challenged through percutaneous exposure of abdomen skin for 20 min in water containing 30 cercariae. Forty-five days post-challenge, adult worms were perfused through their portal veins. Protection was calculated by comparing the number of worms recovered from the immunization group to the control group, using the formula [18]:


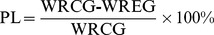


where PL  =  protection level, WRCG  =  worms recovered from the control group, and WREG  =  worms recovered from the experimental group.

To evaluate the liver egg burden, the liver of each mouse was removed, weighed, homogenized, and digested for 30 min at 37°C with 20 mL 10% NaOH. The number of liver eggs per gram was compared to the control mice, using the formula:


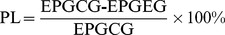


where PL  =  protection level, EPGCG  =  eggs per gram from the control group, and EPGEG  =  eggs per gram from the experimental group.

**Figure 2:**
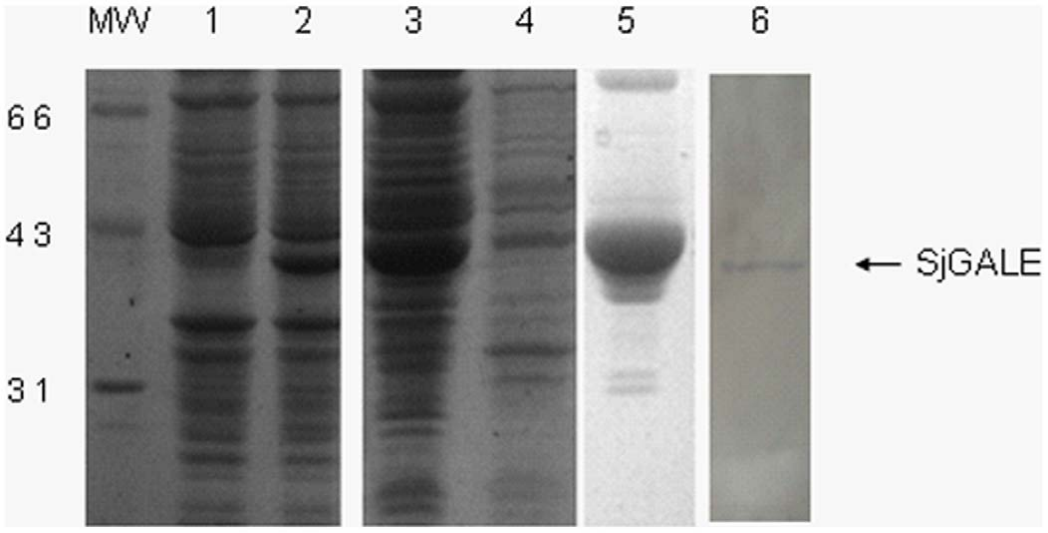
Expression and purification of SjGALE in *E. coli*. Cell extracts and fractions from *E. coli* BL21 (DE3) transformed with the pET28-SjGALE were separated by 12% SDS-PAGE. Lanes 1 and 2: total extract of a clone before and after induction with 1 mM of IPTG. Lanes 3 and 4: supernatant and inclusion bodies after lysis, respectively. Lane 5: the protein in supernatant purified through Ni-NTA His-Bind resin. Lane 6: western blot profile (antigen: native protein of *S. japonicum* and primary antibody: polyclonal anti-rSjGALE antibody).

### 8. Measurement of Anti-SjGALE Antibodies

Following immunization, sera were collected at 0, 15, 30, 45, 60, 75, and 90 days after the first immunization. The measurement of specific anti-SjGALE antibodies was performed using an indirect enzyme-linked immunosorbent assay (ELISA). First, 96-well microtiter plates were coated with 10 µg/mL of rSjGALE for 12–16 h at 4°C, then blocked for 2 h at RT with 200 µL per well of PBS-T plus 1.5% BSA. Then, 100 µL of each sera sample was diluted 1∶100 in PBS-T, added to each well and incubated for 1 h at RT. Plate-bound antibody was detected using peroxidase-conjugated anti-mouse IgG (Sigma-Aldrich, St. Louis, MO, USA), IgG1 (AbD Serotech, Kidlington, UK), and IgG2a (AbD Serotech) diluted in PBS-T to 1∶5000, 1∶2000, and 1∶2000, respectively.

**Figure 3:**
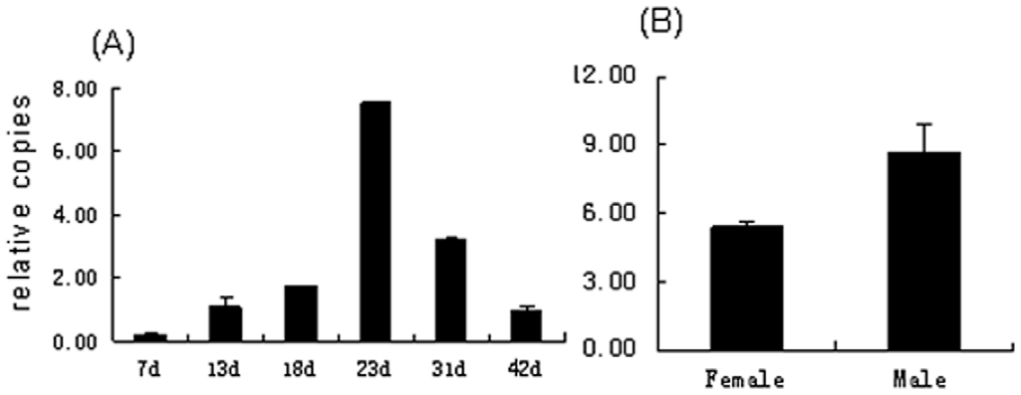
Differential gene expression of SjGALE. Samples (A) throughout six stages of *S. japonicum* and (B) between the male and female adult worms were analyzed by real-time PCR. Data were normalized against amplification of an internal housekeeping control gene (β-tubulin). The data are the means ± SD of one representative of three independent experiments.

**Figure 4:**
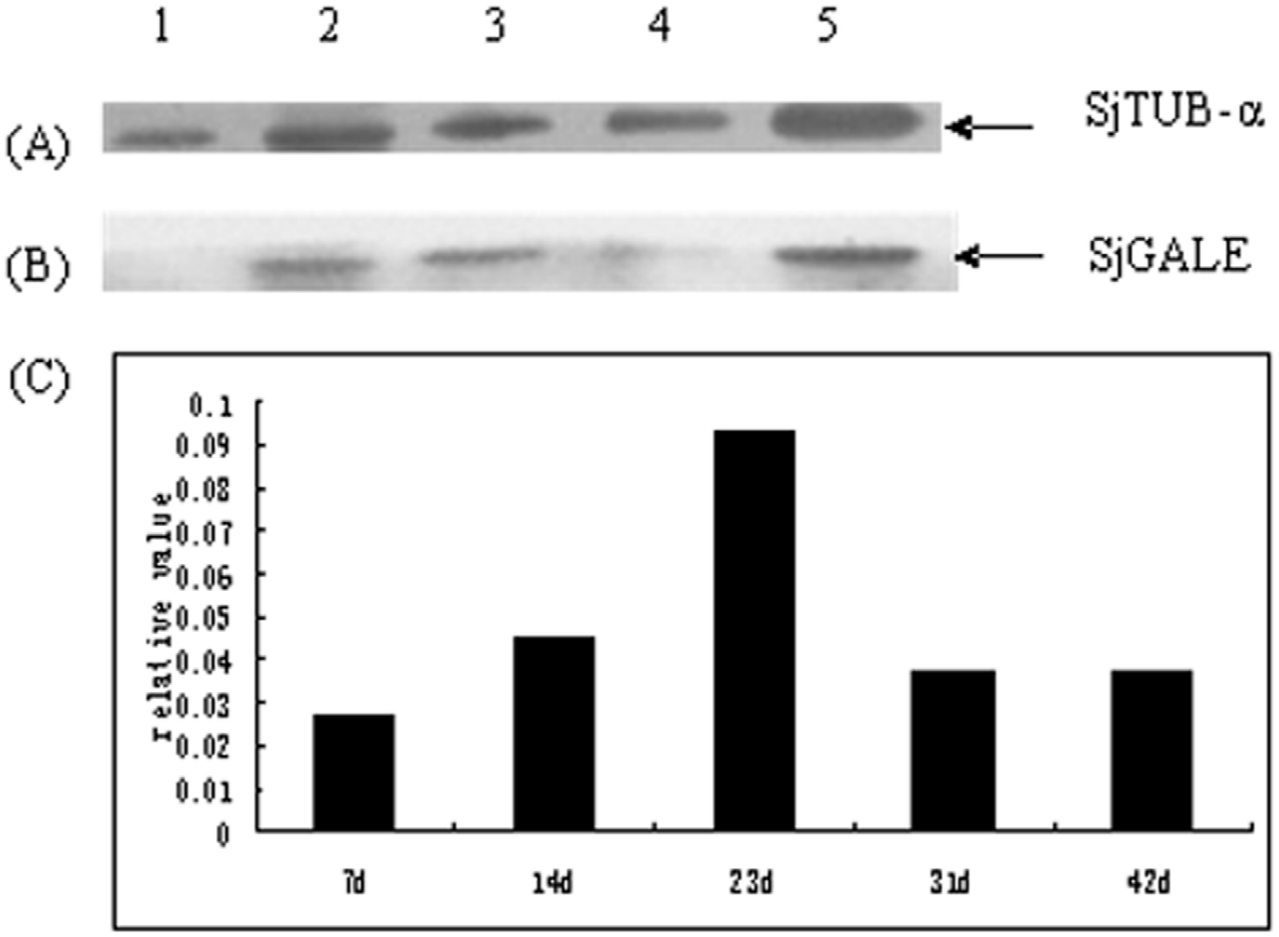
Western blot analysis of the SjGALE protein level at different stages of the *S. japonicum* life cycle. Total protein lysates from different stages of *S. japonicum* as indicated were separated on 12% polyacrylamide gels and electrophoretically transferred to nitrocellulose filters. Blots were probed with mouse anti-SjGALE and anti-α-tubulin as a loading control. (A) Tubulin was used as a control to determine the sample preparation. (B) Extracts were prepared from various stages of the *S. japonicum* life cycle and subjected to immunoblotting with anti-rSjGALE serum. (C) The western blot bands were converted into a histogram by measuring the optic density of the autoradiogram band.

### 9. Cytokine Analysis

Cytokine experiments were performed using splenocyte cultures from individual mice immunized with rSjGALE plus CFA/IFA (n = 5/group). On the 10^th^ day after the third immunization, mice were sacrificed and splenocytes were isolated from the macerated spleens of individual mice using EZ-Sep™ Mouse 1X per the manufacturer’s instructions (Dakewe Biotech Co., Ltd., Shenzhen, China). The cells were washed twice with Roswell Park Memorial Institute (RPMI) 1640 medium (Gibco Life Technologies, Carlsbad, CA, USA) and then adjusted to 1 × 10^6^ cells per well for IL-4, IL-10, IFN-γ, and TNF-α assays supplemented with 10% FBS and 100 U/mL of penicillin-streptomycin. Splenocytes were maintained in culture with medium alone or stimulated with rSjGALE (25 µg/mL). The 96-well plates (Corning Life Sciences, New York, NY, USA) were maintained at 37°C in an atmosphere of 5% CO_2_. For cytokine assays, polymyxin B (30 mg/mL) was added to the cultures, which completely abrogated the cytokine response to LPS as previously described [19]. Culture supernatants were collected after 48 h of rSjGALE stimulation for IL-4 and TNF-α analysis, and also after 72 h of rSjGALE stimulation for IL-10 and IFN-γ analysis [20]. The assays for measurement of IL-4, IL-10, IFN-γ and TNF-α were performed using the Duoset ELISA kit (R&D Diagnostics Ltd., Minneapolis, MN, USA) according to the manufacturer’s instructions.

### 10. Statistical Analysis

Data are expressed as mean ± standard deviation (SD). Statistical analyses were performed using the Student’s *t*-test or one-way ANOVA. A *p*-value <0.05 was considered statistically significant.

**Figure 5:**
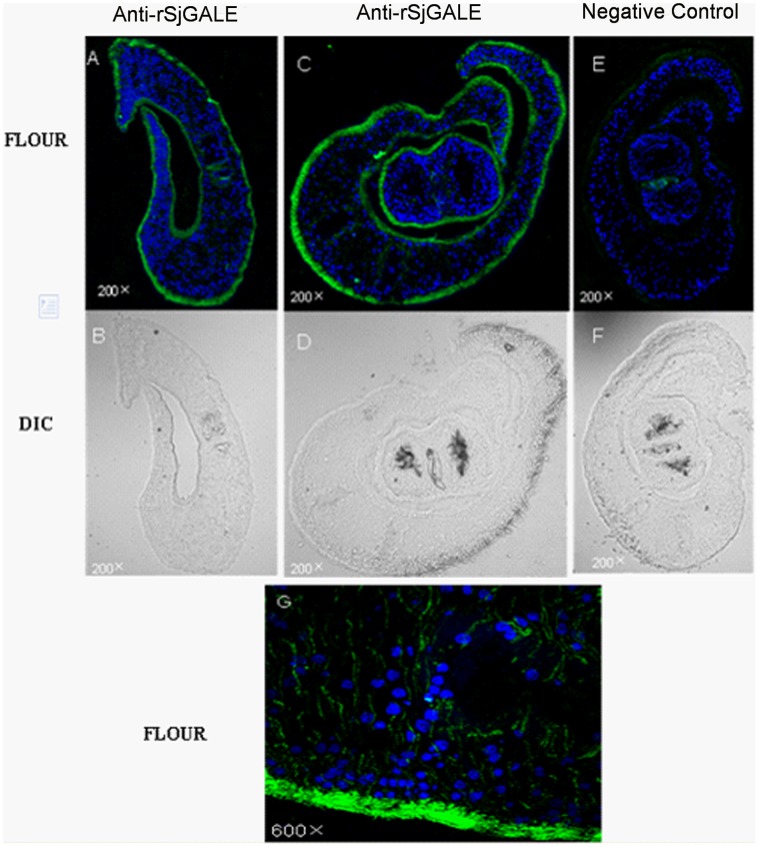
Immunolocalization of SjGALE from adult male and female *S. japonicum*. Polyclonal anti-rSjGALE antibody was used as primary antibody and serum from naive mice was used as a negative control. Alexa Flour 488-conjugated (green) anti-mouse IgG was used as a second antibody. DAPI was used to stain nuclei (blue). (A): male adult worm. (C): male and female paring worms. (E): negative control. (B, D and F): DIC images of A, C, E, separately. (G): Detail of the image from panel (C) for detection of SjGALE (60× magnification).

## Results

### 1. Cloning and Sequence Analysis of SjGALE

The full-length sequence of SjGALE was obtained by PCR amplification with specific oligonucleotides. The sequence displays an ORF of 1056 bp and encodes a protein of 351 amino acids, with a predicted molecular mass of approximately 39.1 kDa and an isoelectric point of 6.06. Post-translational modifications were also predicted and SjGALE did not contain a signal peptide, N-glycosylation sites or transmembrane helices. Comparison of the amino acid sequences showed that the GALE of *S. japonicum* had 89%, 58% and 57% identity with its orthologs in *Schistosoma manosi*, *Mus musculus* and *Homo sapiens*, respectively. The consensus sequence showed that the epimerase domain was conserved among these species (Fig. 1).

**Figure 6:**
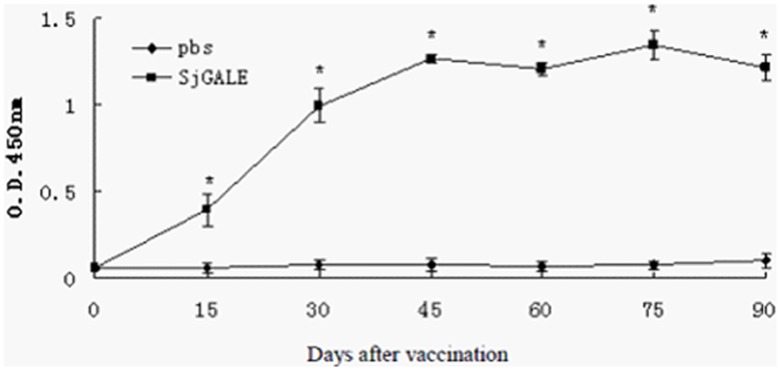
Kinetics of specific anti-SjGALE IgG induced in rSjGALE-immunized mice. Sera of 10 immunized mice per group were collected on days 15, 30, 45, 60, 75 and 90 after the first immunization and analyzed by an ELISA. Results are presented as the mean absorbance of one trial measured at 450 nm for each group of one experiment. Statistically significant differences of vaccinated mice compared to the PBS + adjuvant control group is denoted by one asterisk (*p*<0.05).

### 2. Production of Recombinant SjGALE and Polyclonal Antibody

The SjGALE gene was cloned into the pET28 expression vector, and the protein was expressed in *Escherichia coli* BL21 (DE3) cells induced by IPTG. Protein extract of transformed bacteria contained a ∼40 kDa band (Fig. 2, lane 2). Then, bacteria were extracted using ultrasonic processors and the lysate was separated into soluble and insoluble fractions; the soluble fractions contained the majority of the recombinant protein (Fig. 2, lanes 3 and 4). Next, the soluble protein was purified using nickel-charged columns and the main recombinant protein was pooled in the eluted fractions (Fig. 2, lane 5). After purification, the protein concentration was found to be around 20 mg/mL, which was used to immunize mice for the generation of polyclonal antibodies. The anti-sera were confirmed to recognize the native SjGALE (Fig. 2, lane 6). The polyclonal antibodies were used in immunolocalization assays and western blotting analysis of SjGALE expression levels in *S. japonicum* at different developmental stages.

**Table 1:** IgG1 and IgG2a immune profile induced by vaccination with rSjGALE or PBS.

Days^a^	Groups				
	IgG1		IgG2a		IgG1/IgG2a ratio
	SjGALE	PBS	SjGALE	PBS	SjGALE
15	0.026±0.003	0.031±0.005	0.022±0.002	0.020±0.003	1.20
30	0.612±0.223^b^	0.033±0.008	0.047±0.030^b^	0.035±0.006	12.93
45	1.356±0.237^b^	0.031±0.002	0.137±0.046^b^	0.041±0.007	9.85
60	1.582±0.147^b^	0.043±0.005	0.223±0.151^b^	0.037±0.008	7.11
75	1.574±0.167^b^	0.046±0.009	0.470±0.238^b^	0.034±0.004	3.35
90	1.414±0.155^b^	0.058±0.004	0.521±0.149^b^	0.043±0.008	2.71

a Days after the first immunization.

b Statistically significant compared to group of animals immunized with PBS.

### 3. Transcription Levels and Protein Levels of SjGALE at different Stages of the *S. japonicum* life Cycle

Transcription of SjGALE was evaluated at the mRNA level during the developmental stages of *S. japonicum* using real-time quantitative reverse transcription polymerase chain reaction (qRT-PCR) analysis. Six stages were examined, including 7d, 14d, 18d, 23d, 31d, 42d worms. Meanwhile, female and male worms after 42 days were separated for examination in another experiment. Differences in transcription levels were observed by comparisons to the housekeeping gene, β-tubulin. All samples were run three times in triplicate and the Student’s *t*-test and one-way ANOVA followed by a Tukey pairwise comparison was used to analyze the data.

**Figure 7:**
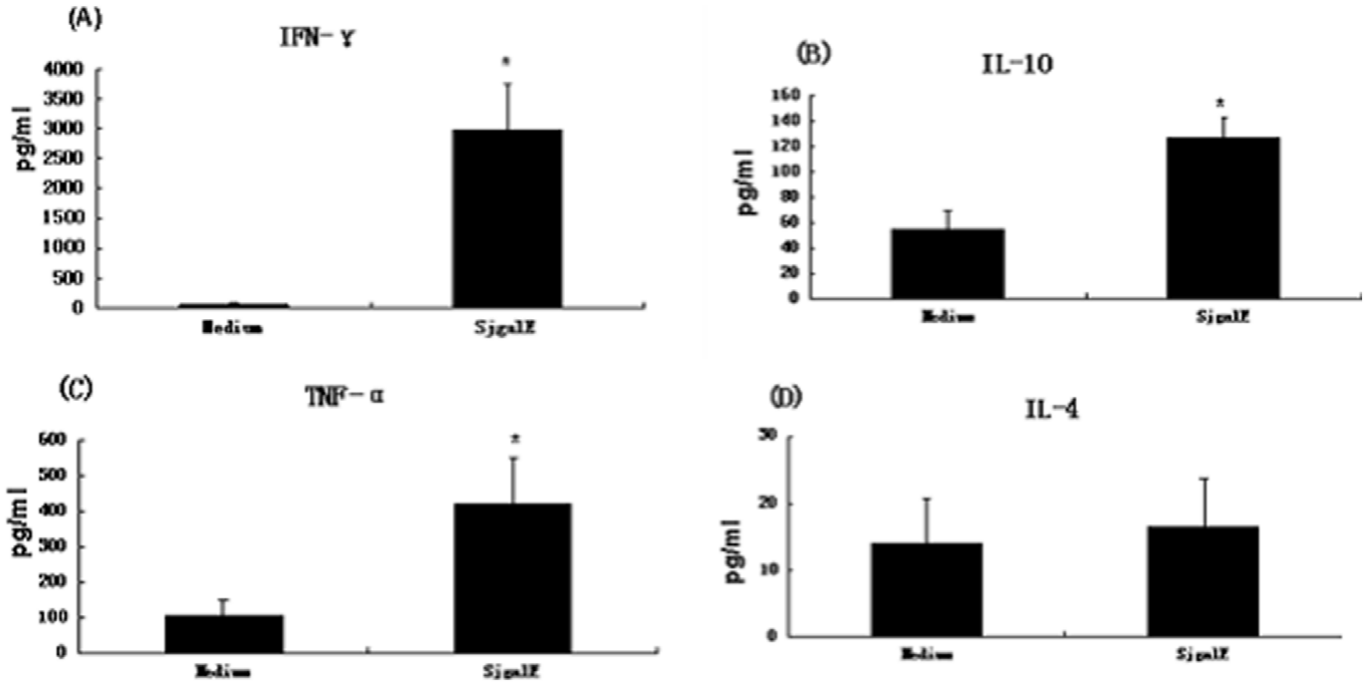
Cytokine profile of rSjGALE-immunized mice. Ten days after the last immunization, splenocytes were isolated and assayed for IL-4, IL-10, IFN-γ and TNF-α production in response to rSjGALE (25 µg/ml) or medium alone, as a control. The results are presented as mean ± SD for each group. Statistically significant differences between cytokines produced after rSjGALE stimulation compared with unstimulated splenocytes are denoted by an asterisk (*p*<0.01).

The results demonstrated that SjGALE mRNA was expressed in all developmental stages examined and exhibited the highest transcription level in 23-day-old worms (Fig. 3A). The results also suggested that the male adult worms expressed a higher transcript level than female adult worms (Fig. 3B). Western blotting was performed on extracts from 7d, 14d, 23d, 31d and 42d worms to evaluate different expression levels of native SjGALE, (Figs. 4A and 4B), then the western blot bands were normalized and converted into a histogram using Image J software (Fig. 4C). The western blot had a similar profile to the qRT-PCR, suggesting a correlation between the transcription level and protein expression. We, therefore, speculated that the native SjGALE had a similar expression trend as the transcription levels.

**Table 2:** Protection level induces by BALB/c mice immunization with rSjGALE plus Freund’s adjuvant and infected with 30 cercariae.

Groups	Adult worms	Liver eggs	Liver eggs/Female adult worm
	Mean±SD	Mean±SD	(% reduction)
	(% reduction)	(% reduction)	
**Trial 1**			
Control			
(PBS+CFA/IFA)	8.8±3.8	28342±15972	9447±5324
n = 10			
SjGALE	5.6±2.9	13324±12379	3188±4536
n = 10	(35.7%* )	(53%**)	(66.2%**)
**Trial 2**			
Control			
(PBS+CFA/IFA)	18.7±5.6	9911±6576	2265±1893
n = 10			
SjGALE	12.5±5.5	5312±2565	916±368
n = 8	(33.0%*)	(46.4%**)	(59.5%**)

* Statistically significant compared to the control group (p<0.05),

** (p<0.01).

### 4. SjGALE is Mainly Located in the Tegument of Adult Worms

An immunolocalization assay, using an antibody specific for rSjGALE, was performed to identify the tissue localization of the SjGALE protein. Results demonstrated that the native SjGALE was mainly distributed over the tegument and at lower levels in the internal tissues of the parasite (Figs. 5A, B, C, D and G); however, no specific staining was observed in sections incubated with naive mouse serum (Figs. 5E and F).

### 5. Antibody Analysis following Immunization

To evaluate the presence of SjGALE-specific IgG, IgG1, and IgG2a antibodies, sera from 10 vaccinated mice from each group were tested using an indirect ELISA. Significant titers of specific anti-SjGALE IgG antibodies were detected at all time points after the first immunization (Fig. 6). In order to determine the IgG isotype profile induced by vaccination, the detection of IgG1 as a Th2 marker and IgG2a as a Th1 marker were also evaluated. The level of SjGALE-specific IgG1 increased rapidly after the second immunization and remained constant after reaching a peak value. In contrast, the IgG2a level increased gradually and the IgG1/IgG2a ratio peaked at day 30 and decreased gradually between days 30 and 90 (Table 1). The reduced IgG1/IgG2a ratio observed in mice immunized with rSjGALE led us to speculate that a Th1 immune response was induced following the vaccination.

### 6. rSjGALE Plus CFA/IFA Immunization Induces a Th1 Cytokine Profile

The cytokine profile produced by splenocytes following rSjGALE stimulation was determined. Polymyxin B was added to the splenocyte cultures to avoid non-specific stimulation due to potential LPS contamination of the purified recombinant protein. Cells from immunized animals stimulated with rSjGALE produced statistically significant amounts of IFN-γ and TNF-α Th1 cytokines, compared to the respective controls cultured with medium alone (Figs. 7A and C). IL-4, the characteristic cytokine of a Th2 immune response, was detected, but was not statistically significantly different between the immunized and control groups (Fig. 7D). Additionally, a significant level of IL-10, which is a regulatory cytokine, was also observed (Fig. 7B). These results indicated that rSjGALE vaccination induced a Th1 cytokine profile.

### 7. rSjGALE Immunization Induces Protective Immunity

To measure the protection levels induced by rSjGALE plus CFA/IFA immunization, mice were challenged with 30 cercariae, and the levels of protection were determined by the number of adult worms recovered in the experimental groups compared to the control group 45 days post-challenge. Mice treated with the recombinant protein followed by challenge infection with *S. japonicum* had lower worm and liver egg burdens (Table 2). Vaccination of mice resulted in a 34% reduction in adult worm burden, a 49% reduction in liver egg burden and a 63% reduction in the number of liver eggs laid per mature female worm (mean values of both trials were combined).

## Discussion

Schistosomiasis continues to be a significant public health concern. An effective control strategy is to develop vaccines to prevent this disease. In recent reports, the use of recombinant antigens, such as Sm-TSP-2 and Sm29, induced ∼50% worm burden reduction, demonstrating that a vaccine to schistosomes is achievable [18], [21], which led us to search for additional vaccine candidates.

In the present study, we characterized SjGALE, not only as a schistosomiasis vaccine candidate, but also other characterizations such as the distribution of SjGALE in adult worms and the differences in transcription and protein levels of SjGALE during the *S. japonicum* life cycle.

GALE is essential for *de novo* biosynthesis of UDP-Gal, a precursor for the biosynthesis of different carbohydrates, glycolipids, and glycosides [11]. Also, SjGALE might play important roles in energy metabolism in *S. Japonicum.* In our study, the SjGALE expression level reached a peak on day 23 (Fig. 3), when the worms achieved the energy needed for egg production, as *S. japonicum* began to lay eggs at this time.

Eggs produced by the worm pairs are important in transmission of the parasite and responsible for pathogenesis [22]. *S. japonicum* discharges 500–3500 eggs/day [23], approximately half of which reach the outside environment by passing through the wall of the intestine and voided with excreta to continue the life cycle, whereas the other 50% are swept into the circulation and are filtered-out in the periportal tracts of the liver resulting in periportal fibrosis, portal hypertension, and the serious sequelae of intestinal schistosomiasis, including hepatosplenomegaly and esophageal and gastric varices [24]. The eggs lodged in the liver are the chief cause of pathology in schistosomiasis [21]; therefore, a reduced egg burden in the liver is a more meaningful endpoint than adult worm burden for evaluating an anti-pathology vaccine. In the present study, we observed reductions of 34% and 49% for mean worm burdens and liver egg burdens, respectively. Although the number of worms and eggs found between the two trials were different for the independence and randomness of the experiment (Table 2), the trend of reduction in worm and liver egg burdens in the two trials was similar. Further, immunization with rSjGALE also affected the reproductive ability of female worms, as the number of eggs laid per female worm in SjGALE-vaccinated mice was reduced by 63% compared to the control animals (Table 2). Immunization with rSjGALE not only reduced parasite load, but also provided prominent protection against host pathology, for it reduced the number of eggs laid by each female worm. More importantly, this immunization had an inhibitory effect on female worm reproduction.

The rSjGALE immunization also elicited a Th1-type immune response, a protective immunization in schistosomiasis. The reduced IgG1/IgG2a ratio (Table 1) following immunization suggested that a Th1 immune response was induced [18]. The cytokine profile (Fig. 7) induced by rSjGALE immunization also improved the Th1-type immune response. rSjGALE immunization induced high levels of IFN-γ, the key cytokine involved in Th1 cell development and low levels of IL-4, the key cytokine involved in Th2 cell development. The involvement of IFN-γ in protective immunity against schistosomiasis is well documented in the murine model [25]. rSjGALE immunization also induced high levels of IL-10, which were found to be regulatory cytokines in many studies and its production is also important in protecting mice against murine schistosomiasis and in the regulation of the granulomatous process [26]–[29].

Immunolocalization studies indicated that SjGALE was mostly located in the tegument of adult *S. japonicum* (Fig. 5). Our colleagues’ studies on the tegument surface protein of *S. japonicum* by biotin labeling found that SjGALE was located and exposed on the surface of worms (data not published), even though SjGALE contains no signal peptide or transmembrane domain (In schistosomes, this is a common phenomena with surface proteins such as Sm21.6 and Sm29). Although there are some similarities between schistosome UDP-glucose-4-epimerase and the human enzyme homolog (Fig. 1), the antigen will not evoke a immune response against the host enzyme because the GALE enzyme exists as a soluble entity in the cytoplasm of the host organism. Many proteins on the tegument of the schistosome have important functions involved in obtaining nutrients from the environment to provide energy [30], [31]. Recently, proteomic research on teguments also identified a number of proteins that may be potential targets for diagnostic tools, drugs, and especially vaccines [32], such as Sm21.6 and Sm29 [18], [20], [33]. Therefore, localization of the SjGALE protein reinforces the potential of this protein as a vaccine candidate.

In conclusion, vaccination of mice with the recombinant SjGALE demonstrated that this protein may be used as a vaccine candidate to control schistosomiasis fecundity and transmission.
